# The LightDock Server: Artificial Intelligence-powered modeling of macromolecular interactions

**DOI:** 10.1093/nar/gkad327

**Published:** 2023-05-04

**Authors:** Brian Jiménez-García, Jorge Roel-Touris, Didier Barradas-Bautista

**Affiliations:** Zymvol Biomodeling, Pau Claris 94 3B, 08010, Barcelona, Spain; Protein Design and Modeling Lab, Department of Structural Biology, Molecular Biology Institute of Barcelona (IBMB-CSIC), Baldiri Reixac 15, 08028Barcelona, Spain; Kaust Visualization Lab, Core lab Division, King Abdullah University of Science and Technology (KAUST), 23955-6900, Thuwal, Saudi Arabia

## Abstract

Computational docking is an instrumental method of the structural biology toolbox. Specifically, integrative modeling software, such as LightDock, arise as complementary and synergetic methods to experimental structural biology techniques. Ubiquitousness and accessibility are fundamental features to promote ease of use and to improve user experience. With this goal in mind, we have developed the LightDock Server, a web server for the integrative modeling of macromolecular interactions, along with several dedicated usage modes. The server builds upon the LightDock macromolecular docking framework, which has proved useful for modeling medium-to-high flexible complexes, antibody-antigen interactions, or membrane-associated protein assemblies. We believe that this free-to-use resource will be a valuable addition to the structural biology community and can be accessed online at: https://server.lightdock.org/

## Graphical Abstract

**Graphical Abstract:**
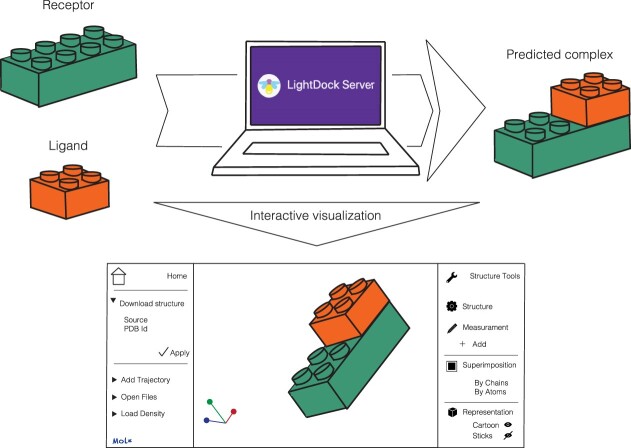
LightDock models macromolecular binary interactions offering an interactive and user-friendly visualization of the results.

## INTRODUCTION

Biomolecular interactions are crucial in cellular environments. In particular, proteins mediate a wide range of molecular processes through their interactions. Progress in computation has paved the way to a better understanding of the role of structure and dynamics in defining the function of these biomolecular systems. All these advances can only be understood with the plethora of experimentally determined structures at atomic resolution deposited at the Protein Data Bank (1) (PDB) and the tremendous advances in experiments over the last decades. Indeed, by leveraging (co)evolutionary information, from multiple sequence alignments (MSAs), and artificial intelligence, AlphaFold2 (2) and RoseTTAFold (3), are showing unprecedented performance in predicting protein structures from sequences. More importantly, they are remarkable at predicting macromolecular complexes (4). Yet, there is still room for improvement in cases where MSAs are limited as antibody–antigen complexes, membrane proteins, or cofactor-mediated interactions (5).

Likely, computational docking might still help in filling this gap and get further insights into molecular association. These classical approaches aim at building three-dimensional models of macromolecular structures by first generating thousands of possible conformations and then discriminating between biologically- and non-biologically relevant poses. With the World Wide Web and all the advances in web software development, it is essential to provide user-friendly web-based services (6) to make these computational approaches more accessible to the overall scientific community. Under this premise, Colabfold (7), the Google Colaboratory-based accelerated implementation of AlphaFold2 and RoseTTAFold, was released, making protein and protein-protein structure prediction accessible to all, among other long-standing web services (8–18). Along these lines, we have developed the LightDock Server, a web-based resource for modeling macromolecular assemblies, with special emphasis on modeling challenging protein-protein interactions.

LightDock is a fully open-source framework for flexible protein–protein, protein–peptide and protein–DNA docking, based on a swarm intelligence optimization algorithm: Glowworm Swarm Optimization (GSO) (19). Swarm intelligence is a family of artificial intelligence algorithms inspired by emergent systems in nature, which perform more efficient searches in complex spaces. In particular, GSO relies on the concept that glowworms feel attracted to each other depending on the quantity of emitted light, a metaphor appropriate for simultaneously capturing multiple local optima in multimodal functions, such as the macromolecular interaction energy landscape. In LightDock, the agents of the GSO algorithm (encoding for receptor-ligand complex poses) are defined as glowworms and carry a luminescent entity called luciferin, which relates to the receptor-ligand interaction energy calculated by the user-selected scoring function(s). After a certain number of optimization steps (100 by default), the generated models typically converge into several clusters, representing the different energy optima of the sampled energetical landscape, which are then ranked according to their score. Here, we present the LightDock Server (https://server.lightdock.org/), an entirely rewritten version of the LightDock framework in the Rust programming language for optimal speed and performance.

## MATERIALS AND METHODS

The server backend has been developed using the Flask Python web micro-framework to process user jobs and to bridge with the LightDock software. The user interface is developed in HTML5 (rendered by Jinja2 template engine) and with NGL Viewer (20) component to display molecular 3D structures. The results page offers interactive and advanced visualization of the generated models powered by Mol* Viewer (21). The LightDock Server runs in a dedicated node with 1 x AMD Ryzen Threadripper 3990X (with 128 effective CPU cores), 96 GB of physical RAM memory, and 7.3TB of disk space in RAID 1 configuration. Thanks to the new implementation of the LightDock protocol in the Rust programming language (https://github.com/lightdock/lightdock-rust, open source released under GPLv3 license), the LightDock Server is capable of running several jobs in parallel with minimum memory footprint.

In the following sections, we report the implementation of the server and describe its potential and applicability by showcasing the modeling of protein-protein interactions under different scenarios,

## RESULTS

### The LightDock server web interface and user experience

**Figure 1.**
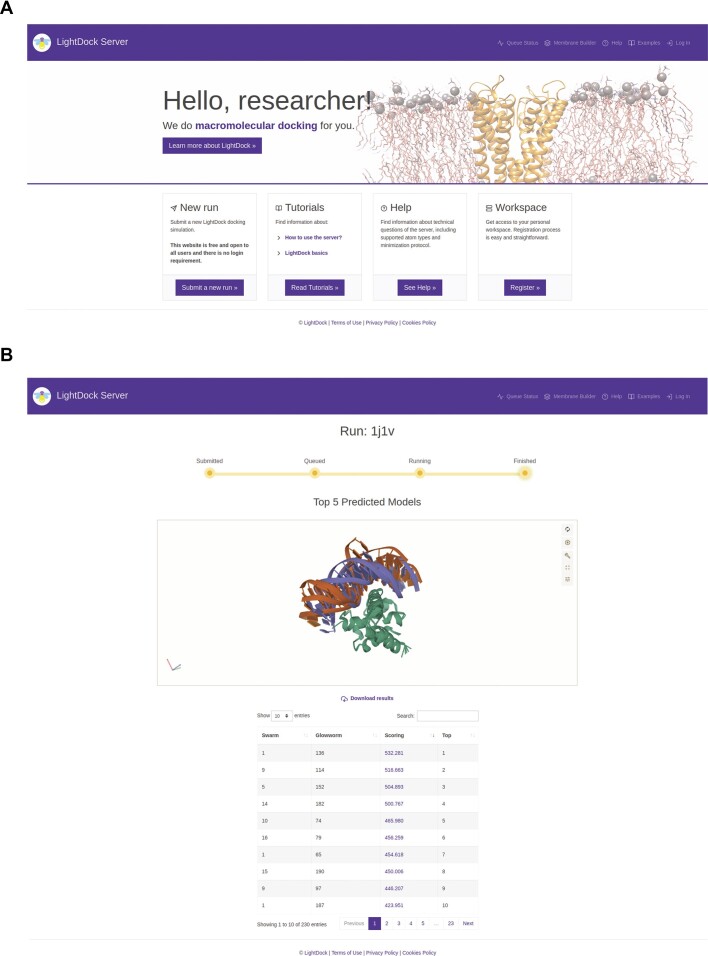
The LightDock Server web interface. The LightDock Server landing page (**A**). A server run example (**B**), this page is automatically updated to reflect changes in the job status and show results when the job is completed.

The LightDock Server is free to use and does not require user registration for any of its predictive modes. The landing page (Figure 1A) provides quick access to the most important sections, i.e. submitting a new simulation (a server job), tutorials, help, or queue status. In general terms, submitting a new simulation is composed of three main steps. In the first step, users are asked to provide a descriptive name for the job and the input receptor and ligand structures in PDB file format. In the second step, users will provide residue-restraints (if any) for receptor and/or ligand partners and select the molecule's nature. Depending on the information in the PDB input files, the LightDock Server will automatically detect some features. For example, if only DNA nucleotides are found in the input receptor structure, the molecule type will be fixed to ‘*DNA*’ for the receptor partner. In the third and final step, users may enable or disable backbone flexibility for any docking partner. Note that for membrane-associated receptors, flexibility is disabled.

Once a job is submitted, the user is redirected to a newly generated view, which is unique for the job and must be bookmarked. This view is regularly updated to provide feedback on the status of the job and, once the simulation is completed (Figure 1B), will display in an interactive 3D web component (Mol* Viewer) the top five predicted structures, together with a dynamic table of the ranking of predicted models, and a link for downloading a compressed file containing the job results. The results view also informs about the date when the job will be removed from the server (2 weeks from the completion date).

**Figure 2.**
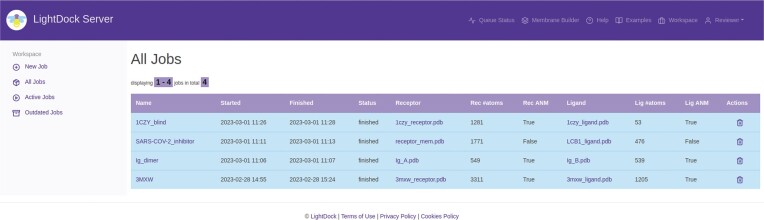
The LightDock Server workspace view. Relevant information about the different user jobs is provided, such as job names, started and finished dates, number of atoms, and direct access to download input structures.

Additionally, registering is possible if users desire to keep track of their jobs for future reference. Once they have registered, users have access to their profile section (where it is also possible to remove their account to fulfill with EU GDPR regulation) and to the *Workspace*, an effective view with four main sections (Figure 2): (i) *New Job* is a link to the job submission interface as described in the previous paragraph, (ii) *All Jobs* shows all jobs submitted by the user, sorted by submission date and with pagination support, (iii) *Active Jobs* shows queued and running jobs submitted by the user and in process by the server and (iv) *Outdated Jobs* view shows jobs submitted by the user with a finish date older than 14 days. Besides the job history, the main advantage of these three last views is the ability to perform special actions on the jobs being processed (stopping them at any point) or on the ones already completed (removing them from the server).

### Overview of the LightDock server functionalities

**Figure 3.**
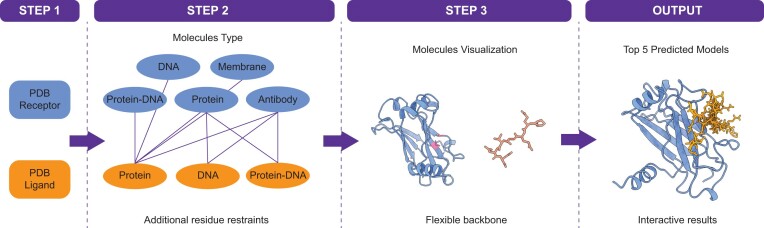
LightDock Server docking simulation workflow. Job submission is divided into three steps. First, the user is requested to upload their molecules and provide a *Job Name*. Second, the molecule type needs to be specified. This step is crucial for activating the desired dedicated modeling mode. Also, additional residue restraints might be provided at this step. Third, the unbound structures and the defined restraints for visual checking will be displayed. At this step, the user might enable flexibility. Finally, the best five models will be interactively displayed for inspection after the simulation.

We have designed dedicated modeling modes depending on the molecule's type and on the absence/presence of information to drive the modeling. Before describing each of them in detail in the following sections, here we provide a number of general considerations. The LightDock Server, which builds upon the LightDock framework, offers the possibility of modeling protein–protein, protein–peptide and protein–DNA interactions. Specifically, and to improve user experience, nine (9) different scenarios are supported (Figure 3) in terms of receptor–ligand molecule's type as follows:

- Protein–protein
- Protein–protein + DNA
- Protein–DNA
- Protein + DNA–protein
- DNA–protein
- Membrane protein–protein
- Antibody–protein
- Antibody–protein + DNA
- Antibody–DNA

When there is no information about the putative interface between two molecules, an exhaustive exploratory docking simulation is an alternative. By default, LightDock generates a dynamic number of *swarms* based on the size and shape of the receptor. However, users may increase the number of initial *swarms* for a denser sampling. When there is information about the putative interface between two molecules, the LightDock Server can incorporate it in the form of residue restraints. These restraints can be specified on the receptor or the receptor and ligand. Please note that restraints on the ligand only are not allowed since this will waste computational resources (note: switch the order of the molecules). If specified on the receptor, the initial *swarms* will be filtered out, and irrelevant sampling regions will be excluded prior to the simulation. If also specified on the ligand, the initial ligand poses will be oriented based on randomly selected receptor-ligand restraint pairs. Besides sampling, the restrained satisfaction rate will bias the scoring during optimization. Once the simulation is completed, the resulting models will be filtered according to the defined restraints, thus assuring that the delivered models agree with the data provided.

Finally, and regardless of the use of residue restraints, the best five or ten poses, according to the score, could undergo a final relaxation step (relaxation protocol is described in the *Help > Minimization* section online) using OpenMM (22) to improve molecular geometry as well as remove potential clashes at the interface. The top five docked complexes will be interactively displayed for visual inspection.

### Dedicated modeling modes

#### Modeling antibody–antigen interactions

Antibodies are known to recognize and bind their antigens through complementary determining regions, most commonly referred to as CDR loops. Several sequence numbering schemes proposed in the literature aim to precisely identify these CDRs without the need to look at the structure. To take advantage of this, we have designed an approach that, given an antibody structure numbered according to a given recognized scheme (Kabat (23), Chothia (24) or IMGT (25)), the server will automatically detect the CDRs and transform them into residue restraints. If the antibody is used as the receptor, the initial swarms will be filtered out so that only those in close proximity to the loops will be kept. The initial poses will be oriented based on random receptor-CDR restraints pairs if used as a ligand. This protocol is compatible with the use of extra information. Since this is a restraint-driven protocol, the resulting models will be filtered out according to the information, and like in the other protocols, the five or ten best docking poses might be further relaxed.

#### Modeling membrane-associated protein assemblies

Membrane protein systems have historically been one of the most difficult systems to study in structural biology. As we have shown, using membrane-derived topological information when modeling the interaction between transmembrane proteins and their soluble partners significantly impacts docking performance. When there is no information about the position of the membrane in the MemProtMD database (26), the user is advised to use our *in-house* protocol (Membrane Builder) to, given an anchor residue, generate an approximated explicit bead membrane mimicking a lipid layer. This preprocessing will enable our membrane-based protocol and the topological information encoded in the membrane will be incorporated into the modeling. It is crucial that the structure provided is in the right orientation so that the generated swarms will be placed in the right position. Additionally, the user could define extra information in both molecules to further narrow the optimization process. As in all restraints-guided protocols, the docked models will be filtered out according to the information provided, with the possibility of a final relaxation.

### Applications of the LightDock server

In this section, we provide a step-by-step guide for modeling a protein-peptide interaction, an antibody-antigen complex, and a membrane-associated protein assembly for demonstrative purposes. All the input files and LightDock results are available at: https://server.lightdock.org/examples

#### Docking the LMP1 binding peptide onto the TNFR-associated factor 2

**Figure 4.**
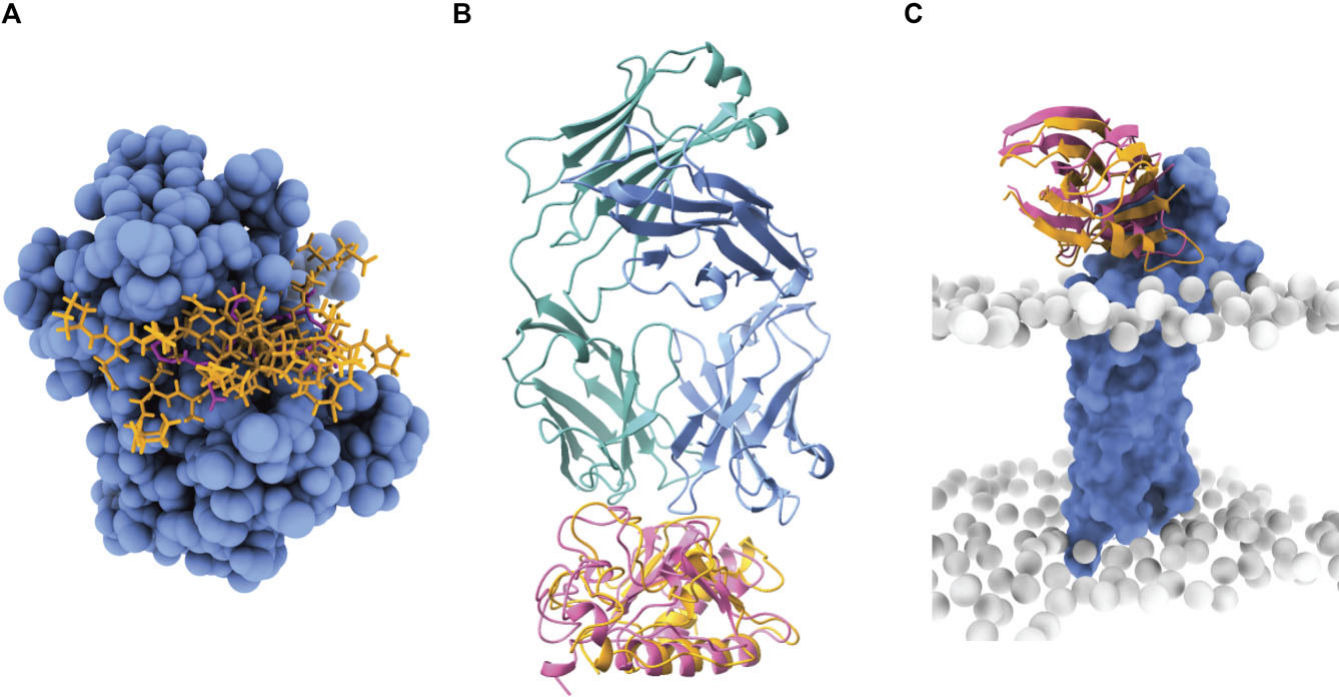
Docking results for each of the described applications of the LightDock Server. Best five LMP1 -TNFR-associated factor 2 (1czy) relaxed models. The receptor is shown as blue spheres while the peptide as orange sticks (**A**). Top anti-Shh-Shh (antibody–antigen) relaxed model resulting from using only sequence-derived information. The antibody is shown in blue, turquoise, and the antigen (Shh) in orange cartoons (**B**). The third docking pose closely recapitulates the 3 × 29 crystal structure (**C**). For comparison purposes, the reference structures are always depicted in pink.

Tumor necrosis receptor-associated factors (TRAFs) can turn on numerous genes involved in inflammatory and immune responses and sustain proliferation during tumorigenesis. TRAFs are constitutively recruited by binding an apparent wide spectrum of peptidic sequences, such as the oncoprotein LMP1, for which the crystal structure was solved over 20 years ago (PDB code: 1czy). For this demonstration, we will use the unbound structure of the receptor from the PeptiDB database (*1czy_receptor.pdb*) and the 7-residue long sequence of the LMP1 peptide (PQQATDD; *1czy_ligand.pdb*) in an extended conformation. Assuming prior knowledge of two interfacial residues on the receptor, we will select A.411 and A.466 as residue restraints with default parameters, flexibility on both molecules and final relaxation of the top 10 models. As shown in Figure 4A, the simulation nicely converges towards the native binding site, thus demonstrating the use of limited information to drive the modeling. The time execution for this case is under a minute.

#### Modeling the trap of the sonic hedgehog metalloprotease by the anti-shh 5E1 antibody

The neutralizing capacities of antibodies are beyond established. However, these proteins have proven key for characterizing molecular mechanisms in biological systems. For example, antibodies can be used for trapping multi-passing transmembrane proteins in distinct conformations to get insights into their functioning. In this example, we will model the interaction between the anti-Shh 5E1 antibody and the Sonic Hedgehog metalloprotease (Shh), a transmembrane protein crucial in cellular differentiation during embryogenesis (PDB code: 3mxw). First, it is important to note that for activating the dedicated antibody mode in the LightDock Server, the antibody must be numbered according to the Chothia scheme and must have chain ids H and L for the heavy and light chains respectively. We will then use this structure (*3mxw_receptor.pdb*) as a receptor and select *Antibody* as molecule type. The Shh protein (*3mxw_ligand.pdb*) will be therefore considered as the ligand (and *Protein* molecule type). In the next step, we will see the residues pertaining to the CDR loops as defined by the numbering scheme highlighted in pink for double-checking. At this step, the user might also introduce extra information such as residue restraints, but we will leave those boxes empty for this demonstration and the remaining parameters as default. Once the simulation finishes, the best five models according to the score will be displayed, and as we can see in Figure 4B, by only using sequence-derived data, the protocol accurately models the interaction as compared to the crystal structure. The time execution for this case is under half an hour.

#### Integrative modeling of claudin-19 in complex with the C-CPE enterotoxin

Claudins are multipass membrane proteins with a major role in cell adhesion. The Clostridium perfringens enterotoxin (C-CPE) can bind to certain claudins, triggering tight junction disintegration and increasing permeability across epithelial cell sheets. It is reasonable to assume that the C-CPE enterotoxin will bind to the extracellular domain(s) of Claudin-19 to disrupt these cell–cell connections. In this example, we will model the Claudin-19–C-CPE (PDB code: 3x29) interaction using membrane topological information from the MemProtMD database, thus targeting the sampling towards the extracellular region.

First, we will browse the 3x29 complex page at MemProtMD, locate the *Data Download* section and download the PDB file corresponding to the coarse-grained (CG) snapshot (MARTINI representation). This file in PDB format contains the MARTINI CG representation of the phospholipid bilayer together with the protein complex. Since we will make use of the phosphate beads as the boundary for the transmembrane region for filtering the sampling region of interest in LightDock, we will remove all atoms except for those representing the PO4 bead and rename them to *BJ* with *MMB* residue name (*3x29_receptor_membrane.pdb*). Given that LightDock performs all-atom docking calculations, we must replace the CG protein with the atomistic one (by simple backbone superimposition; CA to BB). Next, we will retrieve the PDB file of the ligand from the MemCplxDB database (*3x29_ligand.pdb*). To activate the membrane mode, select *Membrane protein* as the molecule type for the receptor, *Protein* for the ligand and enable flexibility on the latter. In this mode, the dynamically generated initial swarms will be filtered to be compatible with the definition of the membrane. As shown in Figure 4C, by using topological information, the protocol recapitulates the crystal structure of the membrane-associated complex. The time execution for this case is four minutes.

Alternatively, when the information is unavailable in the MemProtMD database, we can use sequence-derived information from UniProt (https://www.uniprot.org/uniprot/Q9ET38) and our *in-house* Membrane Builder utility to build a CG bead layer embedding Claudin-19. The topological information from the UniProt entry indicates that it contains four helical transmembrane regions (segments 8–28, 82–102, 118–138 and 161–181) and five extramembranous regions: three cytoplasmic (segments 1–7, 103–117 and 182–224) and two extracellular (segments 29–81 and 139–160). Based on this information, we must select an anchor residue (e.g. S138) and default parameters for execution.

## CONCLUSION AND FUTURE PERSPECTIVES

The LightDock Server is an artificial intelligence-powered service that allows the prediction of macromolecular interactions (polypeptides and ss/dsDNA molecules) in the absence (blind) or presence (driven) of experimental information. Since the original implementation of the LightDock framework, the algorithm showed comparable performance to state-of-the-art docking software (27), with increased predictive capabilities for medium-to-high flexible cases. Recent improvements highlighted its potential to address several biological questions. Enabling information usage derived from experiments, such as hydrogen-deuterium exchange or mutagenesis, into the docking calculations (28) remarkably increased the modeling performance, even with limited and/or partially incorrect data. Our restraints-driven protocol can also incorporate sequence-derived information to model antibody-antigen interactions with excellent results (29). Moreover, the framework allows for the use of explicit low-resolution membranes and accommodates topological information into the modeling of membrane-associated protein assemblies (30). To our knowledge, no other docking software has reported the use of explicit membranes to guide the modeling; thus, this protocol makes LightDock unique.

The LightDock software is in continuous development and improvement. A clear example is that the scientific community is adapting it to a new plethora of scenarios, e.g. the modeling protein–RNA interactions (https://lightdock.org/tutorials/0.9.3/rna_docking) or the rational prediction of PROTAC-compatible protein-protein interfaces (31). As new case scenarios develop and are validated by results, we plan to continuously update the LightDock Server with new dedicated modeling modes and complementary tools. Thanks to its easy-to-use web interface, ubiquity from any modern web browser, and fast delivery of results, the LightDock Server would greatly help to easily test biologically relevant hypotheses, offering distinctive yet validated protocols for the structural biology community.

## DATA AVAILABILITY

The LightDock software is freely available to academic and commercial users (10.5281/zenodo.7706358), including the faster implementation in the Rust programming language (10.5281/zenodo.5883405). LightDock is also distributed via the Python Package Index (https://pypi.org/project/lightdock/). Validation of the software against numerous docking benchmarks as well as comparisons against other algorithms can be found in references 27, 28, 29, 30 and 31, whose results are publicly available and have been also linked at https://server.lightdock.org/help#benchmarking_and_references.
